# Response to “The Importance of Exercising Caution When Comparing Results from Malaria Vaccines Administered on the EPI Schedule and on a Seasonal Schedule”

**DOI:** 10.4269/ajtmh.22-0544b

**Published:** 2022-11-21

**Authors:** Philip J. Rosenthal

**Affiliations:** Department of Medicine University of California San Francisco, California E-mail: philip.rosenthal@ucsf.edu

Dear Sir,

Comments from Drs. Birkett, Miller, and Soisson^1^ providing context for interpretation of results of malaria vaccine efficacy trials that were discussed in my recent article,^2^ are much appreciated. Consideration of published efficacies of the RTS,S and R21 malaria vaccines suggests better efficacy with the R21 vaccine. But, as noted, these data are not truly comparable, and full appreciation of the comparative efficacies of these two vaccines will require additional study.
